# A Simple Repeat Polymorphism in the *MITF-M* Promoter Is a Key Regulator of White Spotting in Dogs

**DOI:** 10.1371/journal.pone.0104363

**Published:** 2014-08-12

**Authors:** Izabella Baranowska Körberg, Elisabeth Sundström, Jennifer R. S. Meadows, Gerli Rosengren Pielberg, Ulla Gustafson, Åke Hedhammar, Elinor K. Karlsson, Jennifer Seddon, Arne Söderberg, Carles Vilà, Xiaolan Zhang, Mikael Åkesson, Kerstin Lindblad-Toh, Göran Andersson, Leif Andersson

**Affiliations:** 1 Department of Animal Breeding and Genetics, Swedish University of Agricultural Sciences, Uppsala, Sweden; 2 Science for Life Laboratory Uppsala, Department of Medical Biochemistry and Microbiology, Uppsala University, Uppsala, Sweden; 3 Department of Clinical Sciences, Swedish University of Agricultural Sciences, Uppsala, Sweden; 4 Broad Institute of Harvard and MIT, Cambridge, Massachusetts, United States of America; 5 FAS Center for Systems Biology, Harvard University, Cambridge, Massachusetts, United States of America; 6 School of Veterinary Science, The University of Queensland, Gatton, Queensland, Australia; 7 Department of Pathology and Wildlife Disease, National Veterinary Institute (SVA), Uppsala, Sweden; 8 Conservation and Evolutionary Genetics Group, Estación Biológica de Doñana (EBD-CSIC), Seville, Spain; 9 Grimsö Wildlife Research Station, Department of Ecology, Swedish University of Agricultural Sciences, Riddarhyttan, Sweden; 10 Department of Veterinary Integrative Biosciences, College of Veterinary Medicine and Biomedical Sciences, Texas A&M University, College Station, Texas, United States of America; Texas A&M University, United States of America

## Abstract

The white spotting locus (*S*) in dogs is colocalized with the *MITF* (microphtalmia-associated transcription factor) gene. The phenotypic effects of the four *S* alleles range from solid colour (*S*) to extreme white spotting (*s^w^*). We have investigated four candidate mutations associated with the *s^w^* allele, a SINE insertion, a SNP at a conserved site and a simple repeat polymorphism all associated with the *MITF-M* promoter as well as a 12 base pair deletion in exon 1B. The variants associated with white spotting at all four loci were also found among wolves and we conclude that none of these could be a sole causal mutation, at least not for extreme white spotting. We propose that the three canine white spotting alleles are not caused by three independent mutations but represent haplotype effects due to different combinations of causal polymorphisms. The simple repeat polymorphism showed extensive diversity both in dogs and wolves, and allele-sharing was common between wolves and white spotted dogs but was non-existent between solid and spotted dogs as well as between wolves and solid dogs. This finding was unexpected as *Solid* is assumed to be the wild-type allele. The data indicate that the simple repeat polymorphism has been a target for selection during dog domestication and breed formation. We also evaluated the significance of the three *MITF-M* associated polymorphisms with a Luciferase assay, and found conclusive evidence that the simple repeat polymorphism affects promoter activity. Three alleles associated with white spotting gave consistently lower promoter activity compared with the allele associated with solid colour. We propose that the simple repeat polymorphism affects cooperativity between transcription factors binding on either flanking sides of the repeat. Thus, both genetic and functional evidence show that the simple repeat polymorphism is a key regulator of white spotting in dogs.

## Introduction

**Figure 1:**
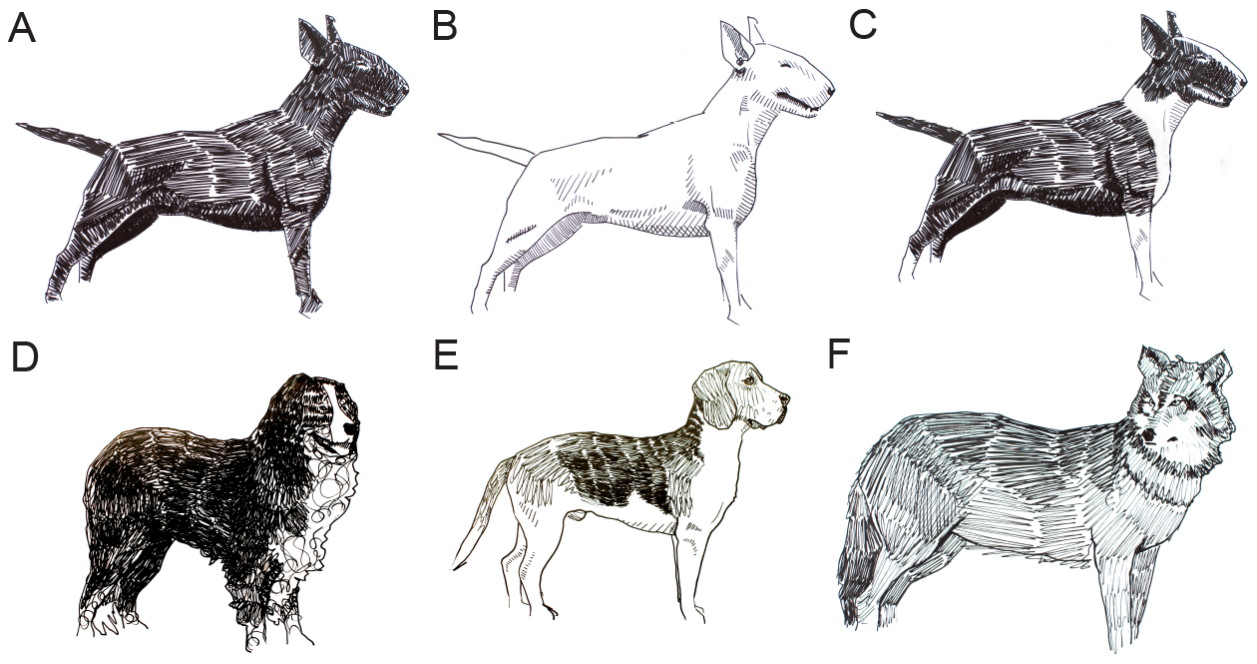
Overview of the different phenotypes included in the study. (A) Solid (*S/S*) Bull terrier, (B) white Bull terrier (*s^w^*/*s^w^*), (C) flash (*S/s^w^*) Bull terrier, (D) Irish spotting (*s^i^*/*s^i^*) in a Bernese Mountain Dog, (E) piebald (*s^p^*/*s^p^*) Beagle, (F) wolf. Drawings: Anders Sundström.

Coat colour variation has fascinated humans for centuries and consequently it has become one of the most extensively studied traits, mainly in mice [1] but also in domestic animals [2], [3]. There is an extensive diversity of white spotting patterns within and between dog breeds caused by genetic factors and stochastic effects during melanocyte development. White spotting indicates an absence of melanocytes in the hair follicles and/or skin due to a failure of melanoblast migration, proliferation or survival during development. In dogs, there is one major white spotting (*S*) locus that was defined by C. Little during the 1950s [4]. He described four different alleles at this locus with phenotypic effects ranging from solid (*S*, Figure 1A), to a completely white coat, caused by homozygosity for the *Extreme white* allele (*s^w^*, Figure 1B). The two intermediate phenotypes were named Irish spotting (*s^i^*, Figure 1D) and piebald (*s^p^*, Figure 1E). Irish spotting is characterized by modest white spotting, often present as a white collar and a white belly, as demonstrated by breeds such as the Bernese Mountain dog and Basenji. Piebald-coloured dogs display limited to extensive white spotting and the phenotype is observed in several breeds, including the Beagle and Fox Terrier. Two *S* alleles are segregating in Boxers: *Solid* (*S*) and *Extreme white* (*s^w^*). These give rise to three different phenotypes: solid (*S/S*), flash (*S/s^w^*, Figure 1C) and extreme white (*s^w^/s^w^*). The flash (*S/s^w^*) phenotype is similar to Irish spotting (*s^i^/s* ^i^) and is therefore often called pseudo-Irish.

In 2006 and 2007, three groups independently mapped some or all of the white spotting phenotypes to the *MITF* (*microphtalmia-associated transcription factor*) locus [5]–[7]. The highest resolution was obtained by Karlsson et al. [6] who mapped the *Spotting* locus to a 1 Mb region on chromosome 20 based on a genome-wide association study comparing 10 homozygous white (*s^w^/s^w^*) and 10 homozygous solid (*S/S*) Boxers. Fine-mapping to a 100 kb region which included *MITF* was achieved by including a second breed, bull terrier, segregating for the same two alleles [6].

*MITF* was an obvious candidate gene because it encodes a transcription factor controlling neural crest-derived melanocyte development and migration [8], [9]. *MITF* has nine alternative promoters that produce multiple isoforms expressed in different tissues. All isoforms share the sequence encoded by exons 2–9, but have unique amino acids in their N-termini, determined by distinct first exons. There is clear conservation across mammals at this gene, as several of the MITF isoforms expressed in human and mouse have also been identified in dog [10], including the melanocyte-specific MITF-M isoform.

To date, 29 mutations in murine *Mitf* have been identified which affect the development and function of melanocytes in the skin, eye and inner ear [1]. Some variants affect vision by reducing eye size (microphtalmia) or cause early onset hearing disorders [11]. In humans, deleterious *MITF* mutations cause disorders of vision and hearing, including the Waardenburg and Tietz syndromes [9], [12], [13]. Deafness has also been recorded in white dogs, where approximately 2% of white dogs (*s^w^/s^w^*) present with bilateral deafness and 18% are unilaterally deaf [14]. The majority of mutations reported in mice and humans that cause severe pleiotropic effects are generally loss-of-function mutations affecting the coding regions [15]. This is not the case with dog *MITF* alleles. A comparison of *S* and *s^w^* haplotypes, using BACs from an *S/s^w^* heterozygote, across the 100 kb canine white spotting candidate region revealed 124 sequence polymorphisms, all of which were located in non-coding regions [6]. This demonstrated that the extreme white coat colour phenotype is controlled by one or several regulatory mutations. This hypothesis is strongly supported by the fact that coloured patches on white spotted dogs display normal pigmentation. Thus, this suggests that the canine *MITF* variants primarily affects migration and survival of melanocytes during development, but have no or only minor effects in mature melanocytes in the hair follicle; pigmentation of the hair requires MITF protein expression [16].

Further analysis of these 124 polymorphisms resulted in a short list of three candidate mutations within or in the vicinity of the *MITF-M* promoter, and one in the *MITF-1B* exon located upstream of *MITF-M*. The first of these is a canine-specific short interspersed nucleotide element (SINEC-Cf element), located about 3 kb upstream of the *MITF-M* transcription start site (TSS). The SINE insertion was only found in dogs presenting the extreme white (*s^w^/s^w^*) or piebald (*s^p^/s^p^*) phenotypes, and was absent in Irish-spotted (*s^i^/s^i^*) and solid (*S/S*) dogs [6]. This very strong association between the SINE insertion and the extreme white and piebald phenotypes was confirmed in a subsequent study based on 324 dogs from 45 breeds although a few exceptions from this rule were noted [17]. The second candidate (SNP#21), a SNP located approximately 1.2 kb upstream of *MITF-M* TSS, occurs in a highly conserved region and the *A* allele at this locus is associated with white spotting alleles [6]. The third polymorphism is a variable length polymorphism (Lp) approximately 100 bp upstream of the *MITF-M* TSS. Long variants of the Lp (LpWhite) are associated with all three white-spotting alleles (*s^w^*, *s^p^* and *s^i^*), whereas all solid dogs examined carried short Lp variants [6], from here on named LpSolid. The fourth candidate mutation, a 12 bp deletion in exon 1B (Exon1B_del) also showed a very strong association with white spotting. It was found on all *Extreme white* and *Piebald* chromosomes tested, but it was also found in the heterozygous state in 4 out of 76 solid dogs. This previous work showed that the *Extreme white* and *Piebald* alleles share the same sequence variants for the SINE, SNP#21, Lp and Exon1B_del polymorphisms, implying that other sequence variants must explain the phenotypic differences between the two alleles.

The aims of the current study were two-fold: (*i*) to screen for the presence of the candidate mutations in wolves that are expected to have the *S* wild-type genotype and (*ii*) to evaluate the functional significance of the candidate mutations associated with the *MITF-M* promoter, alone and in combination. Given the high level of conservation across mammals at this region, any mechanistic insights gained in the canine model could also be used to further understand the role of the master melanocyte regulator, MITF.

## Results

### *MITF* haplotype diversity in wolves

A causal mutation with a major effect on white spotting is expected to be rare or absent in wolves. Wolves show the wild-type coat colour that ranges from dark grey to almost white in arctic populations, but without obvious patches of no pigmentation as is characteristic for dogs carrying the *Extreme white* or *Piebald* alleles. We therefore examined the frequency of the four candidate mutations in a set of 60 wolves with global distribution (Table S1). All tested individuals had a low copy number (CN) status at the *AMY2B* locus (Table S1), known to distinguish wolves (CN = 2) from the majority of domestic dogs (CN = 3–30) [18].

We first screened the wolf samples for the SINE insertion and found that this polymorphism is widespread among both European and North-American wolf populations (Table 1). Thus, we can exclude the possibility that this is a derived mutation in domestic dogs with a major effect on the white spotting phenotype.

**Table 1:** Allele frequencies of three candidate *MITF* polymorphisms in wolves from different geographic regions.

Geographic region	n	SINE^1^	SNP#21*A 2	Exon1B_Del
Scandinavia	34	0.32	0	0.18
Belarus	4	0.50	0	0
Russia	5	0.20	0	0
Bulgaria	2	0.50	0	0
Spain	6	0.08	0.08	0
Italy	1	1.00	0	0
Canada	7	0.21	0.14	0

1 The frequency of the allele with the SINE insertion is presented.

2 The *A* and *G* alleles at SNP#21 are associated with white spotting and solid colour, respectively.

The SNP#21*A allele associated with white spotting in dogs was only found in one Spanish and two Canadian wolves from the 60 wolves tested (Table 1). This suggested that SNP#21 could be a derived mutation in dogs and therefore we screened a larger set of dogs (206 dogs from 17 breeds) with different coat colour phenotypes to assess the strength of association to white spotting (Table S2). This analysis confirmed the association between SNP#21*A and white spotting, but the association was far from complete, since the allele occurred at a high frequency in Irish Wolfhound that has solid colour and it occurred at a low frequency in some breeds showing white spotting (Bearded Collie and Border Collie) (Table S2). We conclude that the allele on its own cannot have a strong effect on white spotting.

The composition (C_X_A_Y_G_2_A_Z_) of the length polymorphism (Lp) in the *MITF-M* promoter makes it very challenging to genotype, as the three mononucleotide repeats show extensive polymorphism as well as length instability during PCR amplification. In a previous study, the composition was determined in dogs using direct sequencing after PCR amplification [6]. In the present study, the repeat and unique flanking sequence was amplified, cloned and the most likely genotype inferred through the sequencing of many clones per individual. In this analysis, we used a conservative approach and so did not designate new alleles unless supported by multiple clones from the same individual. To confirm the utility of this approach we first confirmed the composition of some alleles previously determined by direct sequencing, followed by a screen of 17 wolves (Table 2). The screen revealed an extensive diversity in wolves since as many as 15 alleles were found.

**Table 2:** SINEC-Cf and length polymorphism (Lp) alleles in the *MITF*-*M* promoter among dogs and wolves.

		Lp base composition							
Colour	SINE	C	A	C	A	G	A	Alleles (bp)^3^	Population
**Dogs**									**Dog breed**
Solid (*S*)	−	9	-	-	7	2	11	29A	Yorkshire Terrier^1^
		10	-	-	8	2	11	31A	Various solid dogs^1^^,^ 2
		10	-	-	9	2	11	32A	Golden Retriever, Keeshond^1^
Irish (*s^i^*)	−	13	-	-	8	2	12	35C	Basenji^1^
		14	-	-	8	2	12	36B	Basenji^1^
		14	-	-	8	2	11	**35D**	Bernese Mountain Dog^1^^,^ 2
Piebald (*s^p^*)	+	11	-	-	10	2	12	**35B**	English Springer Spaniel^1^
		12	-	-	10	2	12	36A	English Springer Spaniel, Fox Terrier^1^
White (*s^w^*)	+	11	-	-	7	2	12	**32B**	Dalmatian^1^^,^ 2
		12	-	-	9	2	12	**35A**	Boxer^1^^,^ 2
		11	-	-	10	2	12	**35B**	Bull Terrier^1^
**Wolves**									**Wolf origin**
Wild-type	+	10	-	-	7	2	11	30A	Scandinavia^2^
	+	11	-	-	7	2	12	**32B**	Belarus, Scandinavia^2^
	−	7	1	1	9	2	13	33A	Scandinavia^2^
	−	7	1	1	10	2	13	34A	Scandinavia, USA^2^
	+	11	-	-	10	2	12	**35B**	Scandinavia^2^
	−	7	1	1	10	2	14	35E	Scandinavia^2^
	−	7	1	1	9	2	11	31B	Belarus^2^
	−	10	-	-	8	2	12	32C	Belarus^2^
	−	7	1	1	9	2	12	32D	Spain, Belarus^2^
	−	11	-	-	8	2	11	32E	Spain^2^
	−	12	-	-	8	2	12	34B	Spain^2^
	−	14	-	-	8	2	11	**35D**	Belarus^2^
	−	6	1	3	8	2	12	32F	Canada^2^
	+	12	-	-	9	2	12	**35A**	Canada^2^
	+	12	-	-	7	2	12	33B	USA^2^

1 Determined by direct sequencing in Karlsson *et al.* [6].

2 Determined as the most prevalent clone after cloning PCR products in the present study; only one dog per breed and coat colour type was sequenced.

3 Allele designations are based on the length of the repeat. Alleles with same length but different repeat compositions are distinguished by capital letters. Alleles present in both wolves and dogs are marked in bold.

**Figure 2:**
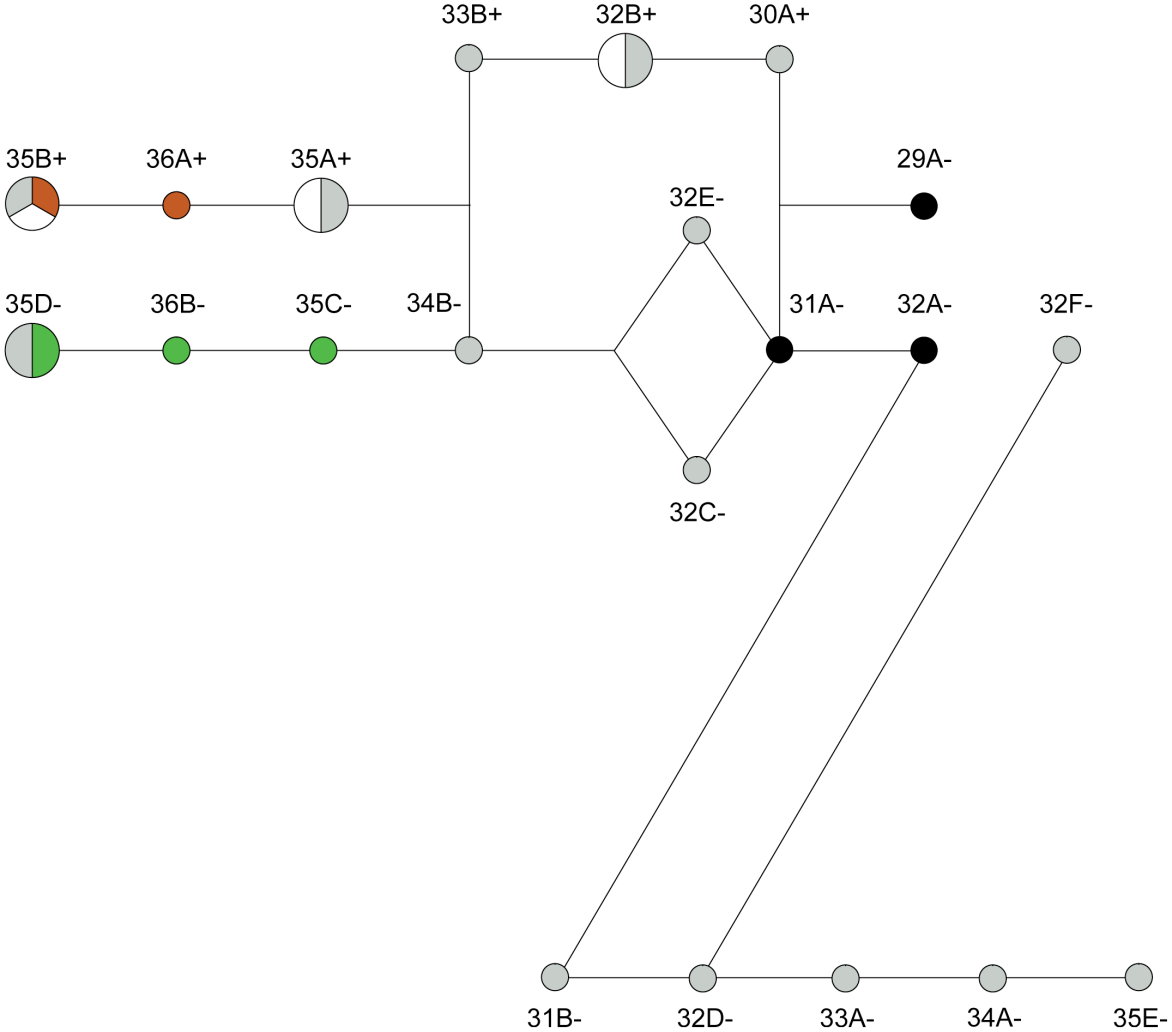
Median joining network for haplotypes generated from the combined SINE and Lp alleles. The designation of Lp alleles is defined in Table 2. + and − after the allele designations indicates the presence/absence of the SINE insertion. Species membership in the network is designated with increasing node size (private<shared). Branch lengths are proportional to the number of mutational steps between the nodes. Colour reflects the white spotting phenotype of the individual sampled (Wolf = grey; for dog: extreme white = white; piebald = brown; Irish = green; solid = black).

We generated weighted conservative median joining network in order to visualize the relationships between the 21 haplotypes generated from the combined SINE and Lp genotypes of dogs and wolves (Figure 2, Table 2). Haplotypes shared between wolves and dogs (i.e. 35B+, 35A+, 35D− and 32B+) are drawn proportionately larger than those that are private (e.g. wolf, 34B−; dog, 35C−). The disrupted C_X_ mononucleotide repeat (C_7_AC or C_6_AC_3_) observed in 50% of wolf Lp alleles is responsible for the long branch, which separates these haplotypes from the torso. This altered motif is yet to be identified in dog (Table 2). Even with this compositional change, the total repeat length was found to be similar between wolves (30–35 bp) and dogs (29–36 bp).

The network was additionally coloured to reflect the white spotting phenotype of the individual sampled. Whilst only six haplotypes contained the SINE element, these included all haplotypes associated with the piebald and extreme white phenotypes. Three of these (32B+, 35A+ and 35B+) were also observed in wolf. Strikingly, a fourth haplotype (35D−) found in wolves was associated with Irish white spotting, in contrast none of the haplotypes found in solid dogs were shared with wolves (Figure 2, Table 2). This result shows that Lp alleles cannot on their own be causal for the extreme white and piebald phenotypes, since we found several wolves sharing the Lp alleles associated with these phenotypes.

The Exon1B-Del polymorphism was screened in all wolf samples and the deletion was only found in wolves from Scandinavia and the allele frequency was estimated at 18% (Table 1). Therefore, this 12 bp deletion could be a derived mutation in dogs, since we cannot exclude the possibility that the presence of the deletion in Scandinavian wolves could be due to dog-wolf hybridization in the past. However, this result also indicates that it is very unlikely that the Exon1B_Del polymorphism is the sole causal mutation underlying extreme white or piebald white spotting, since such phenotypes are not observed in the Scandinavian wolf population.

### Transcriptional activity is affected by the SINE insertion and in particular the length polymorphism (Lp)

**Figure 3:**
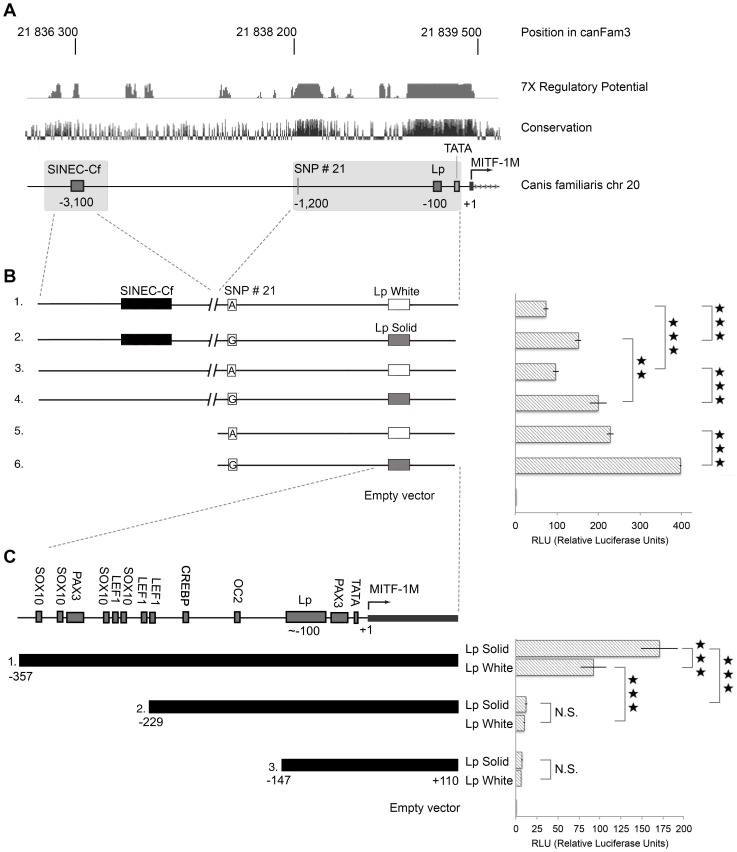
Schematic overview of candidate causative mutations upstream of the canine *MITF-M* promoter and luciferase reporter activity. (A) The SINEC-Cf, SNP#21 and Lp sequences included in constructs are indicated together with a comparative human-dog sequence alignment over the region. Tracks representing 7X regulatory potential and mammalian conservation for the corresponding region in humans are indicated. The broken vertical lines indicate the border between the promoter region and the upstream region combined in individual constructs. The region −1400 to −2800 bp upstream of the *MITF-M* promoter was not included in the construct. (B) Overview of the six luciferase reporter constructs used to assess the regulatory potential of different combinations of the SINE, SNP#21 and Lp variants and results of reporter assays. (C) Critical elements of the canine *MITF-M* promoter. Schematic overview of the canine *MITF-M* minimal promoter. Three different insert fragments (1, 2 and 3) and two variants of each fragment were designed corresponding to the *S* and *s^w^* haplotypes. The insert borders were defined based on the predicted transcription factor binding sites as indicated. Firefly luciferase reporter levels in B and C are presented in relation to control *Renilla* luciferase levels, normalized against the empty control vector. Stars in the graph indicate reporter activity significance levels in pair-wise comparisons; N.S. = Non Significant, * P<0.05, ** P<0.01, *** P<0.001. Error bars represent standard error of the mean. RLU = Relative Luciferase Units.

Luciferase reporter assays in human melanoma 624mel cells were performed to test the functional significance of the region containing the SINE insertion located about 3.1 kb upstream of the *MITF-M* TSS and the Lp located within the *MITF-M* promoter (Figure 3A). Six different reporter constructs were designed: (1) SINE+LpWhite (35A), corresponding to a *s^w^* allele, (2) SINE+LpSolid (31A), (3) no SINE+LpWhite (35A), (4) no SINE+LpSolid (31A), corresponding to a *S* allele, as well as two constructs without the SINE region (5) LpWhite or (6) LpSolid (Figure 3B).

Highly significant differences in transcriptional activity were observed to be associated with the SINE and Lp. In particular, LpWhite was associated with a lower promoter activity in all three matched comparisons with the LpSolid variant (SINE_LpWhite vs. SINE_LpSolid; noSINE_LpWhite vs. no SINE_LpSolid; LpWhite vs. LpSolid; Figure 3B). Constructs containing the SINE insertion were associated with lower luciferase activities in the two matched comparisons with constructs lacking the SINE insertion, but only one reached statistical significance (noSINE_LpSolid vs. SINE_LpSolid; Figure 3B). The two polymorphisms appeared to influence transcriptional activity in an additive manner in this assay, which led to a highly significant, three-fold difference in transcriptional activity between the SINE_LpWhite reporter (mimicking the *Extreme white* haplotype) and the noSINE_LpS reporter (mimicking the *Solid* haplotype).

These constructs were confounded by the relationship between SNP#21 and the Lp since the SNP#21*A allele was always associated with LpWhite and the SNP#21*G with LpSolid (Figure 3B). In order to assess the functional significance of SNP#21, we performed luciferase assays with the constructs SINE_LpWhite, noSINE_LpWhite and LpWhite in which the candidate SNP position was mutated to a G (*S* allele). These constructs were then compared with the originals, which harboured an A at this position. No significant difference in luciferase activity was observed between constructs with the same SINE and Lp alleles differing only with regard to SNP#21 (Figure S1).

### *MITF-M* promoter activity in dogs requires a 128 bp sequence located upstream of the Lp region

The region less than 400 bp upstream of the *MITF-M* promoter harbours eleven known transcription factor binding sites and the corresponding region in humans has an extremely high predicted regulatory potential (Figure 3C). Six different luciferase reporter constructs (Figure 3C) were designed in order to define the elements that constitute the essential parts of the canine *MITF-M* promoter and interact with the Lp variants associated with solid and white coat colour. The results demonstrated that the 128 bp sequence only present in Promoter construct 1, containing four SOX10, one PAX3 and three LEF1 binding sites (Figure 3C), is crucial for *MITF-M* promoter activity. Moreover, the results confirmed that the LpWhite variant is associated with significantly lower activity compared to the solid variant, but only when it occurs in the context of the entire minimal promoter (Promoter 1_LpWhite vs. Promoter 1_LpSolid; Figure 3C).

### Further support for the functional significance of the *MITF-M* length polymorphism

**Figure 4:**
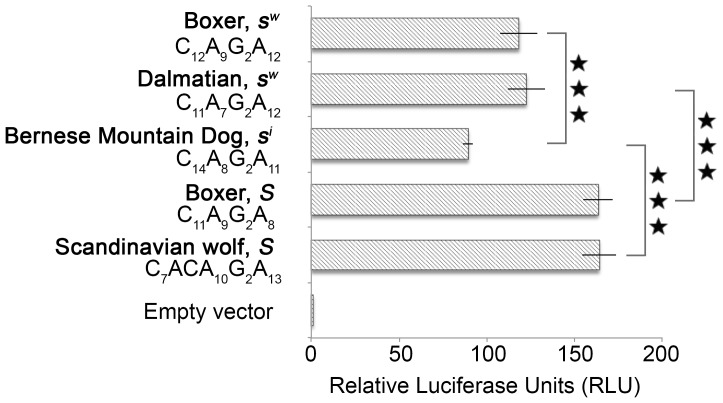
Luciferase activity associated with different alleles of the *MITF* length polymorphism in dogs and wolves. The luciferase constructs were designed according to *MITF-M* minimal promoter activity (fragment 1 in Figure 3C). Firefly luciferase reporter levels are presented in relation to control *Renilla* luciferase levels, normalized against the empty control vector. Stars in the graph indicate reporter activity significance levels in pair-wise comparisons; N.S. = Non Significant, * P<0.05, ** P<0.01, *** P<0.001. Error bars represent standard error of the mean. RLU = Relative Luciferase Units.

Encouraged by the very robust difference in promoter activity between the *Solid* and *Extreme white* Lp variants in the context of four different constructs (Figures 3B and C), we decided to characterize promoter activity with three additional Lp variants using the minimal *MITF* promoter construct described in Figure 3C. Dalmatian dogs are white with black spots and the breeding experiments Little carried out indicated that this spectacular coat colour pattern is due to homozygosity for *Extreme white* and *Ticking* [4]; *Ticking* is a dominant modifier of white spotting that causes spots of pigmented hair in white areas [2]. The *MITF* alleles found in Dalmatians had the shortest Lp (32 bp) of all alleles present in spotted dogs but had a C mononucleotide repeat (n = 11) that was longer than those detected in *Solid* Lp variants, n = 9–10 (Table 2). Interestingly, the Dalmatian Lp construct resulted in a reporter activity indistinguishable from the Boxer *s^w^* variant and significantly lower than the reporter activity associated with the Boxer *S* variant (Figure 4). The Bernese Mountain Dogs exhibit Irish white spotting (*s^i^*/*s^i^*) and carried an Lp variant denoted 35D that was also found in a wolf from Belarus (Table 2). The *s^i^* reporter construct showed even lower promoter activity than the two *s^w^* constructs (Figure 4). Finally, we investigated one representative allele (34A, Table 2) of the Lp motif, characterized by a disrupted C mononucleotide repeat common in wolves but not yet observed among dogs. This construct (C_7_ACAG_2_A_13_) resulted in a reporter activity very similar to the one observed for the solid Boxer variant (Figure 4). These experiments strengthen the association between the Lp and *MITF-M* promoter activity, and show that three different promoter variants associated with a white spotting phenotype are consistently associated with a lower reporter activity than those obtained with constructs mimicking *Solid* alleles.

## Discussion

Coat colour was probably one of the first traits altered during dog domestication. White spotting in dogs can be traced back as long as there are written records or dog portraits, and have been present for thousands of years. Changes in coat colour occur during the domestication due to selection against wild-type colour; as a measure to facilitate animal husbandry (less camouflage), to facilitate the identification of undesired domestic/wild crossbred animals or for fashion and because of relaxed purifying selection [19]. In fact, Columella, the Roman authority on agriculture, wrote already in the first century AD that shepherds prefer white sheep dogs “because it is unlike a wild beast, and sometimes a plain means of distinction is required in the dogs when one is driving off wolves in the obscurity of early morning or even at dusk” [20]. Our previous study demonstrated that the *Extreme white* (*s^w^*) allele in Boxers is not associated with coding changes in *MITF* [6]. This conclusion was achieved by taking advantage of the fact that the reference individual (Tasha) for the dog genome assembly showed the flash phenotype (*S/s^w^*). This allowed us to sequence BACs representing each of the two alleles. A careful examination of the entire non-coding region showing the strongest association to white spotting, combined with a screen across a diverse panel of dogs, revealed four candidate mutations that showed the strongest association to white spotting. The present study demonstrated that none of these four candidates could be the sole causal mutation for *Extreme white*. This is because the four candidate mutations either occur at a relatively high frequency in the wild ancestor of dogs or do not perfectly co-segregate with phenotype among dog breeds. It is possible that these candidate mutations have an effect on pigmentation in wolves and they may even cause some subtle forms of white spotting, as this could be difficult to notice in a wolf with its long fur, basic grey colour and greying with age effect. However, we are convinced that the *Extreme white* allele is not common in wolves, as to the best of our knowledge Extreme white has never been reported in wolves. This colour morph would also be strongly selected against in a natural population due to both the lack of camouflage (except in arctic environments) and the well established association with deafness [14].

Our results combined with the previous characterization of the canine *MITF* locus strongly suggest that the different white spotting alleles (*s^w^*, *s^p^* and *s^i^*) are not caused by three independent mutation events, but rather reflect haplotype effects due to different combinations of multiple causative regulatory mutations selected during dog domestication and breed formation. This interpretation is supported by the observation that there is more extensive sequence similarity among the *s^w^*, *s^p^* and *s^i^* haplotypes than between any of these and haplotypes associated with solid colour [6]. For instance, *Extreme white* and *Piebald* alleles may share all four candidate mutations examined in this study. Thus, *MITF* in dogs is another example of ‘evolution of alleles’ by consecutive accumulation of multiple causal mutations that occurs in domestic animals at loci under strong selection [21].

This study has provided strong genetic and functional evidence that the length polymorphism (Lp) in the *MITF-M* promoter is one of the causal polymorphisms. It is probably the most important regulatory mutation causing decreased *MITF-M* transcription, and consequently affecting white spotting in dogs. Firstly, we have not yet found any overlap between the Lp alleles associated with white-spotting or solid colour in dogs (Table 2). The white-spotting alleles had a longer C mononucleotide repeat and all, except the one found in Dalmatians, had a longer total repeat region. We detected an extensive genetic diversity of the Lp among wolves. We found as many as 15 different alleles when only examining 17 wolves. A very striking and unexpected observation was the considerable Lp allele-sharing between white-spotting alleles in dogs and wolves, but no allele-sharing between solid alleles and wolf alleles. This is unexpected because *Solid* is assumed to be the wild-type allele at this locus. We propose that the explanation for this pattern is that there has been selection both for and against white spotting (thus for solid colour in the latter case) during dog domestication and breed formation. In the early history of domestication there was selection against wild-type colours, while during breed formation there was selection for breed-specific colour such as white spotting in Boxers and solid black colour in Labradors. A repeat polymorphism like this is expected to have a very dynamic evolution, where new alleles are generated by slippage during DNA replication, consistent with the rich Lp diversity among wolves. Thus, the very tight Lp distribution observed in solid dogs and the lack of overlap with alleles found in wolves is hardly in agreement with selective neutrality.

The extensive Lp diversity may have no obvious phenotypic effects in wolves, because such effects may depend on epistatic interaction with other sequence polymorphisms not present in wolves at *MITF* or at other loci in the genome. However, it is also possible that the *MITF* polymorphisms contribute to coat colour diversity in wolves, ranging from dark grey to almost white colour. This is worth exploring using samples from phenotypically well-characterized wolves.

This study also provided functional data supporting the conclusion that the Lp directly affects white spotting. Luciferase reporter constructs containing the longer Lp variants, associated with *Extreme white* (*s^w^*) in Boxers and Dalmatians and with the *Irish spotting* (*s^i^*) allele in Bernese Mountain Dogs, had all significantly lower activities compared with constructs containing the short Lp *Solid* variant, indicating that the Lp affects *MITF-M* transcription (Figures 3B and 4). The *Extreme white* (*s^w^*) and *Solid* Lp variants in Boxers differ in size by four nucleotides, which corresponds to approximately one half turn of the DNA helix. This may have important consequences for the functional interaction between transcription factors binding to either flanking side of the Lp (Figure 3C). Transcription factors that face the same side of the DNA helix in the context of the short Lp *Solid* variant will face opposite sides of the DNA helix in the Lp *White* variant, which is expected to result in reduced capacity to establish functional interaction. Previous studies have shown that transcription is markedly affected when crucial promoter elements are separated by half a helical turn [22]–[23]. In favour of such a scenario is the cluster of LEF1, SOX10 and PAX3 binding sites which are located approximately 200 bp upstream of the Lp. Importantly, the PAX3 and SOX10 transcription factors have been shown to physically interact and to be critical for *MITF-M* transcription [24]. Furthermore, a 10 bp insertion that disrupts a PAX3 binding site in the MITF-M promoter is causing the “splashed white” phenotype in horses [25]. The corresponding PAX3 site in the dog promoter is the one located between the Lp and the TATA-box (Figure 3C). LEF1 is known to physically interact with MITF and to be involved in transcriptional self-activation of the *MITF-M* promoter [26]. Our data support previous findings from other species that the cluster of LEF1, SOX10 and PAX3 binding sites are crucial for *MITF-M* expression (Figure 3C). Our data also demonstrate that it is unlikely that the Lp region has a direct effect on transcription factor binding, as we observed no effect of the Lp variants unless the upstream 200 bp region containing the cluster of LEF1, SOX10 and PAX3 sites were included in the construct.

The SINEC-Cf insertion located about three kb upstream of the *MITF-M* transcription start site had a widespread distribution among wolf populations, implying that this must be an ancestral polymorphism rather than a recent derived mutation. However, the SINE insertion shows a very strong association to the *Extreme white* and *Piebald* haplotypes but is rare or absent among *Solid* and *Irish* spotting haplotypes (those associated with no spotting or the least white spotting). Our reporter assay data suggested that the SINE-region acts as a weak silencer element and that it acts in an additive mode together with the long Lp variants to reduce *MITF-M* promoter activity associated with *s^w^* and *s^p^*. The mechanism underlying the repressive function of the region containing the SINE insertion remains to be defined, but for example, SINE elements are known to be targets for DNA methylation [27]. It is possible that differential methylation within this region is involved in controlling *MITF-M* transcription. Unfortunately, we have not been able to analyse the *MITF-M* expression or methylation status during different stages of canine melanocyte development due to the implicit difficulties in sampling dog embryos.

SNP#21 occurs in the vicinity of an evolutionary conserved element but this actual nucleotide position is not strongly conserved. It could be a derived mutation since it is rare in wolves. The association between SNP#21*A and white spotting across breeds is far from perfect and the reporter assay did not reveal any effect on transcriptional regulation. However, this does not exclude the possibility that this substitution could have a minor effect on white spotting, as a reporter assay will never perfectly replicate transcriptional regulation *in vivo*.

We also provided support for the possibility that the 12 bp deletion in exon 1B is a derived allele that promotes white spotting. This variant was only found in the highly inbred Scandinavian wolves, and was thought unlikely to be causative in the previous study because it was found at a low frequency in some solid dogs [6]. However, the present study has shown that no *MITF* polymorphism shows complete agreement with phenotype and in fact, the insertion in exon 1B, together with the Lp, shows the best concordance with white spotting. The deletion appears to be fixed in dogs with the extreme white and piebald phenotypes, but rare or absent in wolves, solid dogs and dogs with the Irish spotting phenotype. No functional assays were attempted for this polymorphism since it occurs in an exon and in our previous study we concluded that it was unclear if this exon is functional in carnivores [6]. However, recently released RNAseq data (January 2104; Broad CanFam3.1/canFam3; http://genome.ucsc.edu) show that *MITF* exon 1B is transcribed in dogs and was found in one transcript from blood (CUFF.25976.1) and one from lung (CUFF.27047.1) Both transcripts originated from the derived allele associated with *Extreme white* and *Piebald*. This finding suggests that the 12 bp deletion in exon 1B may very well affect MITF function during the development of melanocytes. Thus, the extensive white spotting in Extreme white and Piebald dogs may be due to the combined effect of mutations affecting *MITF-M* transcription and a coding mutation in exon 1B.

The luciferase assay indicated that the Lp variant associated with *Irish spotting* had an even lower *MITF-M* promoter activity than the variants associated with *Extreme white* and *Piebald*, although the two latter alleles cause more extensive white spotting (Figure 4). However, this is consistent with our interpretation that canine *MITF* alleles are not due to single mutations but the combined effect of multiple mutations in the *MITF* region. An important difference between *Irish spotting* and *Extreme white*/*Piebald* is that only the two latter alleles carry the SINE insertion upstream of *MITF-1M* and the 12 bp deletion in *MITF-1B*. Another interesting difference between these two groups of alleles is that *s^i^/s^i^* homozygotes show a high degree of symmetric white spotting whereas *s^p^/s^p^* and *s^w^/s^w^* homozygotes show asymmetric white spotting (Figure 1). This is probably not caused by the nature of the underlying mutations but rather a dosage effect, *i.e.* to which extent MITF function is affected during melanocyte development, because *s^w^/S* heterozygotes also show symmetric white spotting.

*MITF* must be one of the loci in the dog genome that has been under strongest positive selection during domestication and breed formation. It is likely that Little's [4] original classification of three white spotting alleles, *Extreme white*, *Piebald* and *Irish spotting*, is an underestimate and that a more extensive allelic diversity is created by combining the variability at the Lp with other sequence variants in the *MITF* region. For instance, Beagles are considered to be homozygous *Piebald* but as Little described, they vary extensively from almost solid coloured to a phenotype mimicking extreme white. It is still an open question whether this variability is caused by genetic heterogeneity at *MITF* or genetic variation at other loci affecting melanocyte development. In order to advance our knowledge about *MITF* it will be essential to make further genetic studies of dogs that are very well characterized for the white spotting phenotype. We recommend that coat colour is carefully registered when performing genome-wide associations studies of other traits or disorders in breeds with a variable white spotting phenotype, like the Beagle. Such studies will reveal the extent to which the variability in white spotting is caused by genetic heterogeneity at *MITF* or if the influence of yet unidentified modifying loci is at play. Furthermore, it is essential to resequence the entire *MITF* region from *Piebald* and *Irish spotting* chromosomes since they may harbour sequence variants in addition to the ones evaluated in the current study, which were detected in a sequence comparison of the *Extreme white* and *Solid* alleles from a single heterozygous dog [6].

## Materials and Methods

### Design of luciferase constructs

The exact borders for the two genomic regions containing SINE and Lp were chosen based on the 7X regulatory potential and mammalian conservation in the corresponding human regions [28] (Figure 3A). These were identified by performing a BLAT search against the human sequence in the UCSC [29] Genome Browser (http://genome.ucsc.edu/), since the regulatory potential track was only available in the Human Mar. 2006 assembly (NCBI36/hg18). Luciferase reporter constructs were designed as follows (Figure 3B; Table 3): SINE+Lp White (corresponding to the *s^w^* allele), no SINE+LpWhite (similar to *s^i^*), SINE+LpSolid and no SINE+LpSolid (corresponding to *S* allele). In order to investigate the transcriptional activities of the two Lp's in the absence of the SINE-region, two additional constructs were included, with only the long or short Lp, respectively.

**Table 3:** Polymorphisms included in the Luciferase reporter design defined in Figure 3.

	Position			
Sequence polymorphism	Genome assembly (bp)^1^	Position relative to TSS	Phenotype correlation^2^	Designation^2^
Simple repeat (12/14 bases)	21,835,941–21,835,953	−3450 bp	No	25
SINE insertion	21,836,232–21,836,429	−3150 bp	Yes	24
Candidate SNP	21,838,204	−1200 bp	Yes	21
Simple repeat (9 bases)	21,838,718–21,838,745	−700 bp	No	20
SNP	21,838,840	−600 bp	No	19
Length polymorphism	21,839,331–21,839,366	−100 bp	Yes	18
Indel (2 bases)	21,839,397	−60 bp	No	17

The positions of the SINE and length polymorphisms are indicated, as are positions of additional polymorphisms previously considered unlikely to be functionally important [6].

1 Chromosome 20, Broad CanFam3.1, Sept 2011.

2 According to Karlsson *et al.* [6].

Inserts containing SINE/no SINE and LpSolid or White (LpSolid: C_10_A_8_G_2_A_11_, LpWhite: C_12_A_9_G_2_A_12_) (Figure 3B) were PCR-amplified from DNA samples from a solid and a white Boxer using KOD Hot Start DNA Polymerase (Novagen, Merck KGaA, Darmstadt, Germany). Sequences containing the Lp were cloned, transformed and grown according to manufacturer's instructions (pCR®-Blunt II-TOPO® vector, One Shot® MAX Efficiency® DH10B-T1® Competent Cells, Invitrogen, Carlsbad, CA, USA). Purified Lp clones were both sequenced and fragment analysed (MegaBACE, GE Healthcare, Uppsala, Sweden) in order to isolate clones with a correct composition of the Lp. Lp inserts of desired size were subsequently restricted from pCR®-Blunt II-TOPO® vector (Invitrogen), ligated into pGL3 Basic vector (Promega, Madison, WI, USA) and cultured according to manufacturer's instructions (One Shot® MAX Efficiency® DH10B-T1® Competent Cells, Invitrogen), followed by sequencing and verification by fragment analysis to ensure fidelity. PCR products containing the SINE/no SINE were then ligated into the pGL3 Basic+Lp vector. The ready vectors were sequenced and the Lp again verified by fragment analysis. In total, six different constructs were designed (Figure 3B).

The constructs used to identify the canine *MITF* minimal promoter were designed based on the predicted regulatory elements in the region. These were identified by using TransFac Professional and the UCSC Genome Browser 7X regulatory potential and mammalian conservation tracks in the corresponding human region (hg18 assembly). Three different constructs (Promoter 1–3) were designed for both LpSolid and LpWhite (Figure 3C). Promoter 1 was approximately 470 bp, Promoter 2 was approximately 340 bp and Promoter 3 approximately 260 bp. Genomic DNA from *S* and *s^w^* boxers was PCR-amplified, ligated into pGL3 Basic vector (Promega) and cultured according to manufacturer's instructions (One Shot® MAX Efficiency® DH10B-T1® Competent Cells, Invitrogen).

The constructs used to test the different Lp variants were amplified using the same primers as for the Promoter 1 construct. All primers used in PCR and sequencing are listed in Table S3.

### Site-directed mutagenesis

The nucleotide at the candidate SNP#21 located 1.2 kb upstream of the *MITF-M* TSS was altered by site-directed mutagenesis (GENEART Site-Directed Mutagenesis System, Invitrogen) in the following constructs: SINE+LpWhite, no SINE+LpWhite and LpWhite (Figure S1). All constructs were verified by sequencing. Oligonucleotides used in mutagenesis are listed in Table S3.

### Cell culture

The human melanoma cell line 624mel [30] was grown in RPMI 1640 (Gibco, Invitrogen GmbH, Karlsruhe, Germany) supplemented with 10% heat inactivated foetal bovine serum (FBS) (Gibco) and 1X Penicillin, Streptomycin and Glutamine (PSG) (Gibco). Cells were split every two to three days and tested negative for mycoplasma (Venor GeM Mycoplasma Detection Kit, Minerva Biolabs, Berlin, Germany).

### Luciferase assays

Human 624mel melanoma cells [30] were transiently transfected at approximately 90% confluency at passage three in 6-well plates. Triplicates for each construct were performed for each experiment. 2 µg of luciferase reporter plasmid (Promega) and 50 ng of control *Renilla* plasmid (Promega) were transfected into each well of a 6-well plate utilizing 4 µl of Lipofectamine 2000 CD reagent (Invitrogen) in Opti-MEM medium (Gibco). The cells were lysed 24 h post-transfection and Firefly and *Renilla* luciferase activities were measured using the Dual Luciferase Reporter Assay System (Promega) with an Infinite M200 Luminometer (Tecan Munich GmbH, Kirchheim, Germany). Firefly values were divided by *Renilla* values to normalize for fluctuations in plated cells and transfection efficiency. Expression values of all test constructs were compared to the expression of the empty vector. Transfections were independently repeated four times per experiment. Values were transformed utilizing a transformation selector [31] (LN BASE e (X+1)) followed by a one way analysis of variance (Holm-Sidak method) (SigmaPlot v12).

### Genotyping of SINEC-Cf and Length Polymorphism (Lp) in wolves

The presence of the SINEC-Cf insertion was analysed by PCR followed by agarose gel electrophoresis. The Lp and unique flanking sequence from each wolf DNA sample was PCR-amplified and cloned according to manufacturer's instructions (pCR®-Blunt II-TOPO® vector, One Shot® MAX Efficiency® DH10B-T1® Competent Cells, Invitrogen). Approximately 10 colonies/sample were sequenced. Where multiple Lp variants were identified from the same individual, the most prevalent Lp clone was selected to be representative. All primers are specified in Table S3.

### Genotyping of SNP#21 in wolves and dogs

Samples were genotyped on an ABI7900HT instrument using the TaqMan Allelic Discrimination Assay according to the manufacturer's instructions (Applied Biosystems, Foster City, CA, USA). Sequences for primers and probes are available in Table S3.

### *AMY2B* copy number assay

The *AMY2B* locus copy number assay was performed as described [18].

### Haplotype visualisation

The relationship between Lp and SINE haplotypes was investigated with conservative (ε = 0) median network (Network version 4.6.1.1; http://www.fluxus-engineering.com). Haplotype components (Table 2) were weighted (w) as follows: [SINE/-], w = 20; C_X_, w = 10; [-/A], w = 20; [-/C_N_], w = 20; A_Y_, w = 10; A_Z_, w = 10. This generated a priority order where the components with the lowest expected mutation rate (insertion/deletion events) were assigned the highest ranking [32].

## Supporting Information

Figure S1 **Luciferase activity of different combinations of the SINE, LpWhite and the two SNP#21 alleles.** Firefly luciferase reporter levels are presented in relation to control *Renilla* luciferase levels, normalized against the empty control vector. Stars in the graph indicate reporter activity significance levels in pair-wise comparisons; N.S. = Non Significant, * P<0.05, ** P<0.01, *** P<0.001. Error bars represent standard error of the mean. RLU = Relative Luciferase Units.
(JPG)

Table S1 **Wolves genotyped for the SINE, Exon 1B deletion, SNP#21, Lp, and** ***AMY2B*** **copy number.**
(PDF)

Table S2 **Allele frequency for SNP#21 in 17 dog breeds.**
(PDF)

Table S3 **Primer sequences.**
(PDF)
