# Supplementary figures and images for: A Simple Repeat Polymorphism in the MITF-M Promoter Is a Key Regulator of White Spotting in Dogs
Source: PLoS One. 2014 Aug 12;9(8):e104363. doi: 10.1371/journal.pone.0104363 (PMC4130573; doi:10.1371/journal.pone.0104363)

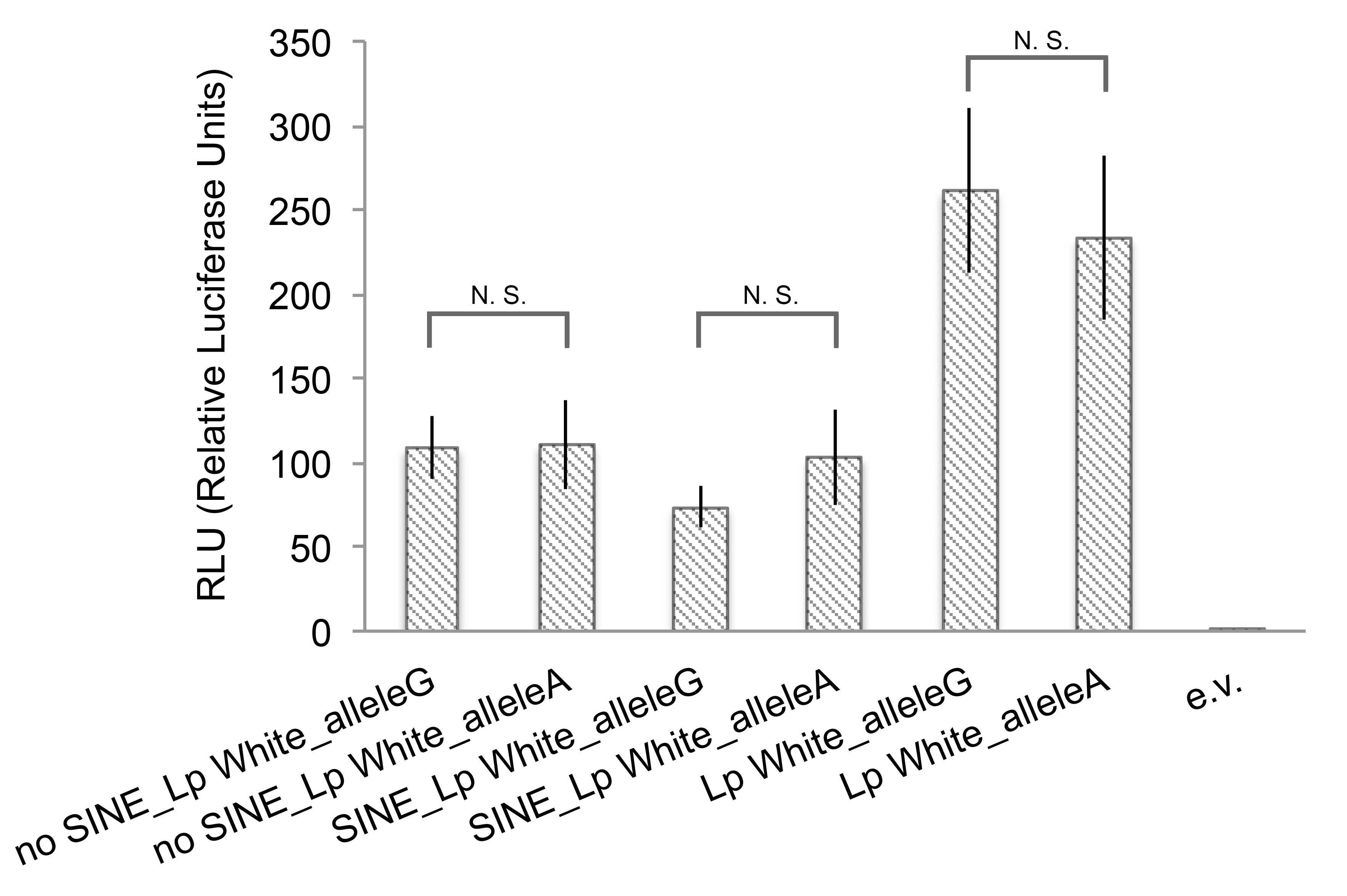

Supplement: Figure S1 — Luciferase activity of different combinations of the SINE, LpWhite and the two SNP#21 alleles. Firefly luciferase reporter levels are presented in relation to control Renilla luciferase levels, normalized against the empty control vector. Stars in the graph indicate reporter activity significance levels in pair-wise comparisons; N.S. = Non Significant, * P<0.05, ** P<0.01, *** P<0.001. Error bars represent standard error of the mean. RLU = Relative Luciferase Units. (JPG) [file pone.0104363.s001.jpg]
