# Supplementary material for: A Simple Repeat Polymorphism in the MITF-M Promoter Is a Key Regulator of White Spotting in Dogs
Source: PLoS One. 2014 Aug 12;9(8):e104363. doi: 10.1371/journal.pone.0104363 (PMC4130573; doi:10.1371/journal.pone.0104363)
Supplement: Table S1 — Wolves genotyped for the SINE, Exon 1B deletion, SNP#21, Lp, and AMY2B copy number. (PDF) [file pone.0104363.s002.pdf]

Table S1. Description of wolf samples and their genotype at *MITF* SINE, exon 1B Length polymorphism (Lp) and SNP#21 and estimated copy number at the *AMY2B* locus.

ID	Origin	SINE	Exon 1B	Lp	SNP#21	AMY2B
SW20	Scandinavia	+/+	88/88	C11A10G2A12	G/G	1.91
SW15	Scandinavia	+/+	100/88	C11A7G2A12	G/G	1.91
SW3	Scandinavia	+/+	100/88	C11A10G2A12	G/G	2.04
SW27	Scandinavia	+/+	100/88	C10A7G2A11	G/G	2.04
SW25	Scandinavia	+/+	100/88		G/G	
SW16	Scandinavia	-/-	100/100	C7ACA10G2A13	G/G	1.85
SW10	Scandinavia	-/-	100/100	C7ACA10G2A13	G/G	
SW9	Scandinavia	-/-	100/100	C7ACA10G2A14	G/G	1.81
SW11	Scandinavia	-/-	100/100	C7ACA10G2A13	G/G	1.98
SW18	Scandinavia	-/-	100/100	C7ACA9G2A13	G/G	2.01
SW30	Scandinavia	-/+	100/100		G/G	
SW32	Scandinavia	-/+	100/100		G/G	
SW1	Scandinavia	-/-	100/100		G/G	
SW2	Scandinavia	-	100/88		G/G	
SW4	Scandinavia	-/+	100/88		G/G	
SW5	Scandinavia	-/+	100/88		G/G	
SW6	Scandinavia	-/+	100/100		G/G	
SW7	Scandinavia	-/+	100/88		G/G	
SW8	Scandinavia	-	100/100		G/G	
SW12	Scandinavia	-/+	100/100		G/G	
SW13	Scandinavia	-/-	100/100		G/G	
SW14	Scandinavia	-/+	100/100		G/G	
SW17	Scandinavia	-/-	100/100		G/G	
SW19	Scandinavia	-/-	100/100		G/G	
SW21	Scandinavia	-/-	100/100		G/G	
SW22	Scandinavia	-/-	100/100		G/G	
SW23	Scandinavia	-/+	100/88		G/G	
SW24	Scandinavia	-/-	100/100		G/G	
SW26	Scandinavia	-/+	100/88		G/G	
SW28	Scandinavia	-/-	100/100		G/G	
SW29	Scandinavia	-/-	100/100		G/G	
SW31	Scandinavia	-	-		G/G	
SW33	Scandinavia	-/+	100/100		G/G	
SW34	Scandinavia	-/+	100/88		G/G	
SW35	Scandinavia	-/-	100/100		G/G	
SW36	Scandinavia	-/-	100/100		G/G	
SW37	Scandinavia	-/-	100/100		G/G	

2W, 22799	Belarus	-/-	100/100	C7ACA9G2A11/C10A8G2A12	G/G	1.78
3W, 22800	Belarus	+/+	100/100	C11A7G2A12	G/G	2.06
4W, 22802	Russia	-/+	100/100		G/G	
5W, 22803	Russia	-/-	100/100		G/G	
6W, 22804	Bulgaria	-/+	100/100		G/G	
7W, 22807	Spain	-/-	100/100	C7ACA9G2A12/C12A8G2A12	G/G	2.09
8W, 22809	Spain	-/-	100/100	C7ACA9G2A12/C11A8G2A11	G/G	2.02
13	Belarus	-/-	100/100	C7ACA9G2A12/C14A8G2A11	G/G	1.81
104	Belarus	+/+	100/100		G/G	
460	Russia	-/-	100/100		G/G	
520	Russia	-/+	100/100		G/G	
523	Russia	-/-	100/100		G/G	
719	Bulgaria	-/+	100/100		G/G	
JAT1	Spain	-/-	100/100	C7ACA9G2A12/C12A8G2A12	G/G	2.34
CVA426	Spain	-/-	100/100		G/G	
A34	Spain	-/+	100/100		G/A	1.94
A39	Spain	-/-	100/100		G/G	
WCA1	Canada	-/+	100/100	C6AC3A8G2A12/C12A9G2A12	G/A	1.99
WCA2	Canada	-/-	100/100		G/G	
WCA3	Canada	-/-	100/100		G/G	
WCA4	Canada	-/-	100/100		G/G	
WCA5	Canada	-/-	100/100		G/G	
WCA6	Canada	-/+	100/100		G/G	
WCA7	Canada	-/+	100/100		G/A	2.04
W36	Italy	+/+	100/100		G/G	
8	China	-/+	?		G/G	
9	China	-/-	?		G/G	
WUSA1	USA	-/+	?	C7ACA10G2A13/C12A7G2A12		
Jordan	Jordan	-/+	?			
