# Supplementary material for: A Simple Repeat Polymorphism in the MITF-M Promoter Is a Key Regulator of White Spotting in Dogs
Source: PLoS One. 2014 Aug 12;9(8):e104363. doi: 10.1371/journal.pone.0104363 (PMC4130573; doi:10.1371/journal.pone.0104363)
Supplement: Table S2 — Allele frequency for SNP#21 in 17 dog breeds. (PDF) [file pone.0104363.s003.pdf]

Table S2. Frequency of the A allele at the *MITF* SNP#21 associated with white spotting in dogs

Breed	Colour (genotype)[1]	n	pA
Beagle	Piebald (s^p/s^p)	15	0.80
Bearded collie	Irish (s^i/s^i)	15	0
Border collie	Irish (s^i/s^i)	15	0.17
Boxer	White (s^w/s^w)	5	1.00
	Flash (S/s^w)	1	0.5
	Solid (S/S)	5	0
Bull terrier	White (s^w/s^w)	6	1.00
English springer spaniel	Piebald (s^p/s^p)	13	0.92
Giant schnauzer	Solid (S/S)	15	0.03
Great Dane	Solid (S/S)	3	0
Hovawart	Solid (S/S)	16	0.03
Irish wolfhound	Solid (S/S)	16	1.00
Labrador	Solid (S/S)	8	0.06
Leonberger	Solid (S/S)	8	0
Newfoundland	Solid (S/S)	14	0.43
Polish lowland	Piebald (s^p/s^p)	8	0.81
Shar pei	Solid (S/S)	14	0.71
Swedish elkhound	Solid (S/S)	13	0.19
Welsh springer spaniel	Piebald (s^p/s^p)	16	0.94

[1]The indicated colour and expected genotypes are based on the most common colour pattern in each breed except for those breeds (Boxer and Bull terrier) that are segregating for colour; the indicated colour for Boxers and Bull terriers are based on individual records.
