# Supplementary material for: A Simple Repeat Polymorphism in the MITF-M Promoter Is a Key Regulator of White Spotting in Dogs
Source: PLoS One. 2014 Aug 12;9(8):e104363. doi: 10.1371/journal.pone.0104363 (PMC4130573; doi:10.1371/journal.pone.0104363)
Supplement: Table S3 — Primer sequences. (PDF) [file pone.0104363.s004.pdf]

Table S3. Primers and probes used for the characterization of the canine *MITF* locus.

| **Name** | **Primer sequence 5'-3'** |
|---|---|
| Luciferase Construct PCR primers | |
| SineF_NheI | GCGTCGACGCTAGCTGAAATTGATGGAAAATAACCTG |
| SineR_Bgl2 | GTCGACGCAGATCTCTCGGGGCTCTCTGACTTT |
| LpF_Bgl2 | GCGTCGACAGATCTATACATTTCACTCTGATTACC |
| LpR_Hind3 | GTCGACGCAAGCTTTATTCACTCTCTCTACTTTCTG |
| F_PromMITF_1_Bgl2 | GCGTCGACAGATCTTCTTCTCCAAAGGGGCAGC |
| F_PromMITF_2_Bgl2 | GCGTCGACAGATCTGCTCATCACTTAAAAAGATT |
| F_PromMITF_3_Bgl2 | GCGTCGACAGATCTTGAAGACCAAGCTCGTAGGA |
| R_PromMITF_Hind3 | GTCGACGCAAGCTTTCCAATGAGAAACGGTAGAC |
| Mutagenesis oligos | |
| Mutagen_S_F | ATTTATTTTTAAATGTGTAGCCCTTTCTTTTTTAAGTGTA |
| Mutagen_S_R | TACACTTAAAAAAGAAAGGGCTACACATTTAAAAATAAAT |
| Sequencing primers | |
| SINE_seq_Fprimer | GTTCTCATTTATATGCTTGC |
| SINE_seq_Rprimer | GTAAGACCCTAAACCCTTT |
| Lp_seq_Fprimer | GTGAGTTTGACTTTGATAGC |
| Lp_seq_Rprimer | AGACTGACTGGCATAGAGA |
| RV3F (RVprimer3) | CTAGCAAAATAGGCTGTCCC |
| GL2R (GLprimer2) | CTTTATGTTTTTGGCGTCTTCCA |
| Fwd_con_mitf1 | CAGGATCTGAATGAAGAAAG |
| Fwd_con_mitf2 | TCACAATATGGCTGTGAG |
| Fwd_con_mitf5 | TGCTTAAGATAGCTCACCA |
| Fwd_con_mitf6 | TCATGTGTAATAGTAGCTTTTAGG |
| Rev_con_mitf1 | AGTGCTTGAGTATTATTACCAC |
| Rev_con_mitf3 | AGGAGGTGGAACGTCTCTA |
| Rev_con_mitf4 | TTACTCATTTACTTGTTCCATTA |
| Rev_con_mitf5 | AGTGATGAGCTATCAAAGTCA |
| Fragment analysis primers | |
| MITF_LpFR_F | TGAAGACCAAGCTCGTAGGAC |
| MITF_LpFR_R_FAM | FAM - GACTGGCATAGAGAAGGCACT |
| SINE insertion primers | |
| SINE_MITF_F | TGGGTGGTTAGTTTGAAGGTC |
| SINE_middle_F | CCCTCTGCCTATGTCTCTGC |
| SINE_MITF_R | CCCCATCTTAGCCATGCTATT |
| Exon 1B primers | |
| 1B_F | AGGATGCTCCTCGGAACTG |
| 1B_R | CTCCGAAATGCAAACGAAAG |

| SNP#21 TaqMan Genotyping Assay | |
|---|---|
| F | GGGCAATGGGTGTAATGTGTTTATTT |
| R | AAAGAATAAGGATCTGCCAGTGAATTAACA |
| Probe_allele_G | VIC-TTTAAATGTGTAGCCCTTTC-NFQ |
| Probe_allele_A | FAM-TTTTAAATGTGTAACCCTTTC-NFQ |
